# The quality of reporting of primary test accuracy studies in obstetrics and gynaecology: application of the STARD criteria

**DOI:** 10.1186/1472-6874-11-8

**Published:** 2011-03-23

**Authors:** Tara J Selman, R Katie Morris, Javier Zamora, Khalid S Khan

**Affiliations:** 1 School of Clinical and Experimental Medicine (Reproduction, Genes and Development), University of Birmingham, Birmingham Women's Hospital, Birmingham, B15 2TG, UK; 2 Clinical Biostatistics Unit, Hospital Ramón y Cajal, Madrid, Spain

## Abstract

**Background:**

In obstetrics and gynaecology there has been a rapid growth in the development of new tests and primary studies of their accuracy. It is imperative that such studies are reported with transparency allowing the detection of any potential bias that may invalidate the results. The objective of this study was to determine the quality of reporting in diagnostic test accuracy studies in obstetrics and gynaecology using the Standards for Reporting of Diagnostic Accuracy - STARD checklist.

**Methods:**

The included studies of ten systematic reviews were assessed for compliance with each of the reporting criteria. Using appropriate statistical tests we investigated whether there was an improvement in reporting quality since the introduction of the STARD checklist, whether a correlation existed between study sample size, country of origin of study and reporting quality.

**Results:**

A total of 300 studies were included (195 for obstetrics, 105 for gynaecology). The overall reporting quality of included studies to the STARD criteria was poor. Obstetric studies reported adequately > 50% of the time for 62.1% (18/29) of the items while gynaecologic studies did the same 51.7% (15/29). There was a greater mean compliance with STARD criteria in the included obstetric studies than the gynaecological (p < 0.0001). There was a positive correlation, in both obstetrics (p < 0.0001) and gynaecology (p = 0.0123), between study sample size and reporting quality. No correlation between geographical area of publication and compliance with the reporting criteria could be demonstrated.

**Conclusions:**

The reporting quality of papers in obstetrics and gynaecology is improving. This may be due to initiatives such as the STARD checklist as well as historical progress in awareness among authors of the need to accurately report studies. There is however considerable scope for further improvement.

## Background

In obstetrics and gynaecology there has been a rapid growth in the development of new tests and primary studies of their accuracy. These studies generate a comparison of the result from an index test against an accepted reference standard [1]. The accuracy of the index test is usually expressed as sensitivity and specificity or other measures like the diagnostic odds ratio (DOR), likelihood ratio (LR) or area under a receiver-operator characteristics curve [2]. These allow clinicians to judge the usefulness and suitability of testing in clinical practice. It is imperative that such studies are reported with transparency allowing the detection of any potential bias that may invalidate the results [3-5]. Guidelines for the reporting of other study types have widely been accepted e.g. CONSORT [6] for randomised control trials. There has been a format for reporting evaluations of tests called Standards for Reporting of Diagnostic Accuracy - STARD [7], introduced in 2003.

The object of the STARD initiative is to improve the reporting of test accuracy studies to allow for the detection of potential bias in a study and to make a judgement on the applicability of the index test results. One of the benefits of using the STARD initiative is to develop a consistent reporting format across all types of tests. The STARD group identified 33 previously published checklists for diagnostic research. From an initial 75 point check list a consensus meeting formulated a 25 point list that could be employed to accuracy studies. This list was designed to help readers judge the studies and to act as a study design tool for authors. Points were specifically chosen on evidence supporting their ability to show variations in measures of diagnostic accuracy [7]. Further supplementing the checklist was flow diagram which aids the assessment of the study population, the recruitment method and indicates the numbers receiving the index test, those excluded and those compared with the reference standard at different stages of the study. STARD should allow a reader to critically appraise the study design, analysis and results.

Previous studies have looked at the impact of STARD in specific clinical areas [8-12] with varying outcomes and the overall quality of reporting of studies which was generally found to be poor. Smidt et al studied reporting quality pre and post STARD in twelve general medical journals and found the mean number of STARD items reported pre-STARD publication was 11.9 (3.5-19.5) and post-publication was 13.6 (4.0-21.0). Coppus et al found that the mean compliance for articles published in *Fertility and Sterility *and *Human Reproduction *was 12.1 (6.5-20). There is no published research looking at the impact of STARD in obstetrics.

This study aims to assess the reporting quality of test accuracy studies in obstetrics and gynaecology and the impact of the STARD statement and compare the quality between the two specialities.

## Methods

We developed a protocol to assess the impact of STARD on studies included in ten systematic reviews performed over the period 2004-2007. The studies covered the time period 1977-2007. We included reviews of minimal and non invasive tests to determine the lymph node status in gynaecological cancers [13-15] and reviews of Down's serum screening markers and uterine artery Doppler to predict small for gestational age in obstetrics [16,17]. These systematic reviews were selected as they were all performed by the authors according to prospective protocols and recommended methodology with prospective assessment of reporting quality using the STARD checklist thus uniform assessment could be ensured. The STARD checklist was applied to each of the studies included in all the reviews with the reporting item being determined as either present, absent, unclear or not applicable (additional file 1). All studies were assessed by TJS and RKM in duplicate, where there was disagreement consensus was achieved following assessment by a third reviewer (KSK). In the event that several tests had been applied to the same patient, the results including the largest number of patients were used in this study or where there was no difference, one index test was selected at random, this ensured patients were only included once.

We addressed the following questions: Has the introduction of STARD improved reporting quality?; does study size correlate with reporting quality?; is there a geographical pattern to reporting quality?. The percentage compliance of studies with STARD items was compared between both specialties before and after the introduction of STARD and over time using the unpaired t test to assess the effect of STARD on the reporting quality of studies. With the publication of STARD in 2003 the assumption was made that all studies published pre 2004 were published without the benefit of this directive.

We examined the relationship between sample size and compliance with STARD using Spearman's rank correlation coefficient (Rho). Kruskal Wallis was used to investigate any relationship between geographical distribution and reporting quality. The country of origin of a study was determined by the country of the corresponding author. Where a significant result was found, pairways comparison was made using Conover Inman procedure. Countries were grouped depending on the number of articles published and the mean journal impact factor and adjusted for gross domestic product and population, based on previous publication [18]. Where there was a large disparity in number of studies per geographical area, some studies were re grouped to avoid large differences in group size and potentially spurious results. For obstetric reviews geographical areas were Oceania, USA, Canada, Asia, Japan, Africa, Eastern Europe and Western Europe and for gynaecology studies there were no studies from Oceania or Canada, but Latin America was added.

In the initial analysis those reporting items coded as unclear and not applicable were excluded. For all of the above analysis, due to the uncertainty of whether reporting items coded as unclear represented methodological failure, sensitivity analysis was performed excluding this code and adding it to the not reported group for all comparisons. Similarly sensitivity analysis was also performed to assess the effect of those items assessed as not applicable, with their initially exclusion to the analysis and then addition as if they were reported so as not to penalise studies which had a larger number of not applicable items and would therefore potentially have a seemingly lower compliance with STARD.

## Results

A total of 300 studies (195 obstetric and 105 gynaecological studies) were identified and included in this analysis. 82% (160/195) of the obstetric and 83.8% (88/105) of the gynaecological studies were published prior to the STARD initiative. The overall percentage compliance with individual reporting items and percentage compliance pre- and post-STARD publication is shown in table 1 for gynaecology and table 2 for obstetrics. The included obstetric studies reported adequately > 50% of the time for 62.1% (18/ 29) of the items as assessed in this review and for gynaecology 51.7% (15/29). Items where reporting was uniformly poor (both obstetrics and gynaecology studies < 50%) were participant sampling, description of technique of reference standard, description of expertise of people performing index and reference standard, blinding of results of index test to those interpreting reference standard, assessment of test reproducibility, tabulation of results and description of adverse events.

**Table 1:** Percentage compliance with individual STARD criteria for included diagnostic accuracy studies in gynaecology

STARD Item	Description	Percentage compliance overall gynaecology (%) N = 105	Percentage compliance pre 2004 (%) N = 85	Percentage compliance post 2004 (%) N = 17	Difference % (95% CI)
S1	Article is identified as study of diagnostic accuracy	64.8%	64.8%	64.7%	-0.1% (-25%; 25%)
S2	States research question or aims	63.6%	63.6%	64.7%	1.1% (-24%; 26%)
S3	Describes study population	77.3%	77.3%	64.7%	-12.6% (-35%; 10%)
S4	Describes participant recruitment	35.2%	35.2%	94.1%	58.9% (33%; 85%)
S5	Describes participant sampling	40.9%	40.9%	64.7%	23.8% (-2%; 50%)
S6	Describes index standard	83.0%	83.0%	88.2%	5.3% (-14%; 24%)
S7	Describes reference standard	30.7%	30.7%	76.5%	45.8% (21%; 71%)
S8a	Describes technique of index test	50.0%	50.0%	82.4%	32.4% (7%; 58%)
S8b	Describes technique of reference standard	30.7%	30.7%	70.6%	39.9% (15%; 65%)
S9a	Describes cut-off for index test	96.6%	96.6%	70.6%	-26.0% (-40%; -12%)
S9b	Describes cut-off for reference standard	0.0%	0.0%	0.0%	0.0% (0%; 0%)
S10a	Describes persons executing index test	3.4%	3.4%	5.9%	2.5% (-7%; 12%)
S10b	Describes persons executing reference standard	0.0%	0.0%	0.0%	0%
S11a	Were results of index test blinded?	22.7%	22.7%	0.0%	-22.7% (-43%; -2%)
S11b	Were results of reference test blinded?	20.5%	20.5%	11.8%	-8.7% (-29%; 12%)
S12	Describes methods for statistics used	30.7%	30.7%	0.0%	-30.7% (-53%; -8%)
S13	Describes methods for calculating test reproducibility	0.0%	0.0%	0.0%	0%
S14	Reports dates of study	86.4%	86.4%	94.1%	7.8% (-9%; 25%)
S15	Reports characteristics of study population	10.2%	10.2%	11.8%	1.5% (-14%; 17%)
S16	Reports number of eligible patients that did not undergo either test	34.1%	34.1%	41.2%	7.1% (-18%; 32%)
S17	Time interval between tests and any treatment	70.5%	70.5%	94.1%	23.7% (1%; 46%)
S18	Reports distribution of severity of disease	75.0%	75.0%	94.1%	19.1% (-2%; 41%)
S19	Reports cross tabulation of results	42.0%	42.0%	29.4%	-12.6% (-38%; 13%)
S20	Reports adverse events	15.9%	15.9%	17.6%	1.7% (-17%; 21%)
S21	Reports estimates of diagnostic accuracy	62.5%	62.5%	41.2%	-21.3% (-47%; 4%)
S22	Reports how missing results were handled	21.6%	21.6%	35.3%	13.7% (-8%; 36%)
S23	Reports estimates of variability of accuracy	0.0%	0.0%	0.0%	0%
S24	Reports estimates of test reproducibility	0.0%	0.0%	0.0%	0%
S25	Discuss clinical applicability of findings	100.0%	100.0%	100.0%	0%

* 100% not applicable

**Table 2:** Percentage compliance with individual STARD criteria for included diagnostic accuracy studies in obstetrics

STARD Item	Description	Percentage compliance overall obstetrics (%) N = 105	Percentage compliance pre 2004 (%) N = 85	Percentage compliance post 2004 (%) N = 17	Difference % (95% CI)
S1	Article is identified as study of diagnostic accuracy	27.2	19.4%	62.9%	43.5% (27%; 60%)
S2	States research question or aims	94.9	93.8%	100.0%	6.3% (-2%; 14%)
S3	Describes study population	74.4	72.5%	82.9%	10.4% (-6%; 26%)
S4	Describes participant recruitment	85.1	83.8%	91.4%	7.7% (-5%; 21%)
S5	Describes participant sampling	36.4	33.1%	51.4%	18.3% (1%; 36%)
S6	Describes index standard	59.5	58.8%	62.9%	4.1% (-14%; 22%)
S7	Describes reference standard	86.7	85.0%	94.3%	9.3% (-3%; 22%)
S8a	Describes technique of index test	45.1	45.0%	45.7%	0.7% (-17%; 19%)
S8b	Describes technique of reference standard	0	0.0%	0.0%	0%
S9a	Describes cut-off for index test	96.9	96.3%	100.0%	3.8% (-3%; 10%)
S9b	Describes cut-off for reference standard	75.9	75.0%	80.0%	5.0% (-11%; 21%)
S10a	Describes persons executing index test	8.2	9.4%	2.9%	-6.5% (-17%; 4%)
S10b	Describes persons executing reference standard	0	0.0%	0.0%	0%
S11a	Were results of index test blinded?	100	100.0%	100.0%	0%
S11b	Were results of reference test blinded?	8.2	6.3%	17.1%	10.9% (1%; 21%)
S12	Describes methods for statistics used	53.3	48.1%	77.1%	29.0% (11%; 47%)
S13	Describes methods for calculating test reproducibility	12.3	13.8%	5.7%	-8.0% (-20%; 4%)
S14	Reports dates of study	65.1	63.1%	74.3%	11.2% (-6%; 29%)
S15	Reports characteristics of study population	67.2	61.3%	94.3%	33.0% (16%; 50%)
S16	Reports number of eligible patients that did not undergo either test	69.2	69.4%	71.4%	2.1% (-15%; 19%)
S17	Time interval between tests and any treatment	11.8	10.0%	20.0%	10.0% (-2%; 22%)
S18	Reports distribution of severity of disease	86.7	83.8%	100.0%	16.3% (4%; 29%)
S19	Reports cross tabulation of results	49.2	48.8%	51.4%	2.7% (-16%; 21%)
S20	Reports adverse events	0*	0.0%	0.0%	0%
S21	Reports estimates of diagnostic accuracy	54.4	50.0%	74.3%	24.3% (6%; 43%)
S22	Reports how missing results were handled	63.6	63.1%	65.7%	2.6% (-15%; 20%)
S23	Reports estimates of variability of accuracy	56.4	50.6%	82.9%	32.2% (14%; 50%)
S24	Reports estimates of test reproducibility	12.8	14.4%	5.7%	-8.7% (-21%; 4%)
S25	Discuss clinical applicability of findings	99.5	100.0%	97.1%	-2.9% (-5%; 0%)

* 100% not applicable

**Figure 1:**
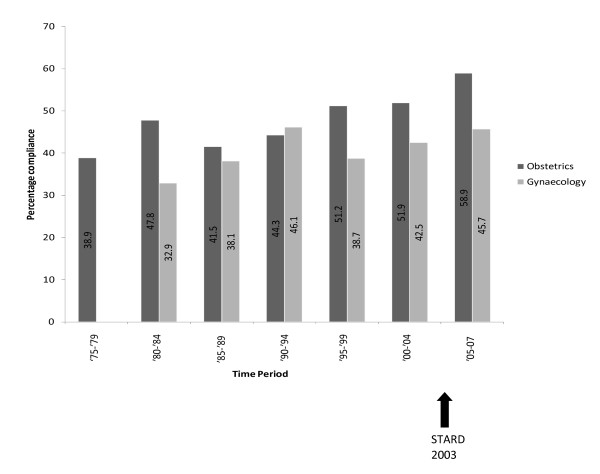
**Bar chart showing mean percentage compliance of studies with STARD criteria, line shows trend over time**.

There was a greater compliance with STARD for obstetric than gynaecological studies (p = < 0.0001). There was significant improvement in the reporting quality of obstetric studies after the introduction of STARD (p = 0.0004). Two studies in obstetrics used a STARD flow diagram following the publication of STARD. Although there was an improvement in the mean compliance in gynaecological studies as well, this did not reach significance (p = 0.08). Tables 1 and 2 also demonstrate the mean differences in percentage compliance pre and post-STARD publication. Figure 1 shows the trend in compliance with the STARD criteria over time. Analysis of the correlation between sample size and compliance with STARD revealed a positive correlation in both obstetrics (Rho = 0.37, p = < 0.0001) and gynaecology (Rho = 0.24, p = 0.0123). Investigation in to the relationship between geographical area of publication and the compliance with STARD showed no relationship for obstetrics or gynaecology, (Kruskal-Wallis 5.05 p = 0.65 and 6.79 p = 0.24 for obstetrics and gynaecology respectively) table 3. Sensitivity analysis showed no significant difference in any of the results.

**Table 3:** Mean percentage compliance of studies with STARD according to geographical area of publication

Area of publication	Mean percentage compliance obstetrics (%) [number of studies]	Mean percentage compliance gynaecology (%) [number of studies]
Africa	66.7% [1]	No studies
Asia	49.3% [8]	41.1% [12]
Canada	56.3% [8]	No studies
Eastern europe	51.7% [16]	39/3% [5]
Japan	50% [7]	47.5% [4]
Latin America	No studies	45% [2]
Oceania	56.3% [8]	33.3% [1]
United States of America	49.7% [44]	38/7% [29]
Western Europe	49.1% [104]	42.4% [52]

## Discussion

The reporting of included studies in this review overall was poor with obstetric studies demonstrating better reporting than the gynaecological studies. In both specialties the geographical origin had no effect on the reporting quality; however the study size showed a positive correlation. There has been a trend in improvement in reporting quality, more so in obstetrics than gynaecology, however there is still significant room for improvement.

There was poor compliance with STARD in many of the studies in this review, in many studies it was unclear whether the study complied with the reporting item. This lack of clarity could potentially affect our inferences, but in other fields it is well known that unclear reporting is associated with bias [19]. Although the studies crossed both obstetrics and gynaecology they were limited to a subset of conditions within these fields. It is likely that these results can be translated across obstetrics and gynaecology, however care should be taken as to the generalisability of this study.

We compared our results to those from similar studies in other subject areas. Within reproductive medicine the reporting of individual items still showed wide variation post STARD publication^8^. In medical journals there was an improvement post STARD in the reporting of calculating test reproducibility, distribution of severity of disease, variability in accuracy between subgroups and use of a flow diagram^9^. In obstetrics and gynaecology there was an improvement in describing participant sampling/recruitment, description/blinding of reference standard, reporting the characteristics of the study population, distribution of severity of disease and variability of accuracy. There was no significant improvement in the use of a flow diagram. In gynaecology however, there were some items that showed poorer reporting post STARD such as description of cut-off of index test and blinding. There are thus no particular items of the STARD checklist that have been poorly adopted or interpreted by authors more that authors have been slow to adopt the STARD checklist and with its publication still being very recent. As more journals adopt the STARD statement and more authors make use of it at the planning and data collection stage of their research there will hopefully be a considerable improvement in the reporting quality in all subject areas in the future.

Poor reporting of a study does not necessarily correlate with bad quality. Accurate reporting is necessary to allow transparency of a study and to ensure the results are interpreted correctly. The application of the STARD checklist may help prevent the implementation of unnecessary or inaccurate tests which can lead to unnecessary financial expenditure and potentially serious consequences for patients.

## Conclusion

The reporting quality of papers in obstetrics and gynaecology is improving. This may be due to initiatives such as the STARD checklist as well as historical progress in awareness among authors of the need to accurately report studies. There is however considerable scope for further improvement.

## Competing interests

The authors declare that they have no competing interests.

## Authors' contributions

The following authors were responsible for study concept and design: TJS, RKM, JZ, KSK. TJS and RKM take responsibility for acquisition of data. All authors were responsible for analysis, interpretation of data, drafting of the manuscript, critical revision of the manuscript and statistical analysis. All authors confirm that they have read and approved the final manuscript.

## Pre-publication history

The pre-publication history for this paper can be accessed here:

http://www.biomedcentral.com/1472-6874/11/8/prepub

## Supplementary Material

Additional file 1 **STARD checklist**. This file contains the Standards for Reporting of Diagnostic Accuracy checklist and a description of each of the checklist items. Click here for file
