# Supplementary material for: The quality of reporting of primary test accuracy studies in obstetrics and gynaecology: application of the STARD criteria
Source: BMC Womens Health. 2011 Mar 23;11:8. doi: 10.1186/1472-6874-11-8 (PMC3072919; doi:10.1186/1472-6874-11-8)
Supplement: Additional file 1 — STARD checklist. This file contains the Standards for Reporting of Diagnostic Accuracy checklist and a description of each of the checklist items. [file 1472-6874-11-8-S1.DOC]

**Supplemental file S1:STARD CHECKLIST**

| **Section and Topic** | **Item** |  | **Code**  **1 2 3 4** |
| --- | --- | --- | --- |
| **TITLE, ABSTRACT AND KEYWORDS** | | | |
|  | **1** | Identify the article as a study of diagnostic accuracy (recommend MeSH heading “ sensitivity and specificity”) | **Yes □ No □ Unclear □ N/A □** |
| **INTRODUCTION** | | | |
|  | **2** | State the research questions or aims, such as estimating diagnostic accuracy or comparing accuracy between tests or across participant groups | **Yes □ No □ Unclear □ N/A □** |
| **METHODS** | | | |
| **Participants** | **3** | Describe the **study population**: the inclusion and exclusion criteria and the settings and locations where the data were collected. | **Yes □ No □ Unclear □ N/A □** |
|  | **4** | Describe **participant recruitment**: was this based on presenting symptoms, results from previous tests, or the fact that the participants had received the index tests or the reference standard? | **Yes □ No □ Unclear □ N/A □** |
|  | **5** | Describe **participant sampling**: was this a consecutive series of participants defined by selection criteria in items 3 and 4? If not, specify how participants were further selected. | **1= consecutive 2=random**  **3= unclear 4=N/a** |
|  | **6** | Describe data collection: was data collection planned before the index tests and reference standard were performed (prospective study) or after (retrospective study)? | **1= prospective 2= retrospective**  **3= unclear 4= N/a** |
| **Test Methods** | **7** | Describe the reference standards and its rationale. | **Yes □ No □ Unclear □ N/A □** |
|  | **8** | Describe technical specifications of material and methods involved, including how and when measurements were taken, or cite references for a) index test or b) reference test | **Yes □ No □ Unclear □ N/A □** |
|  | **9** | Describe definition of and rationale for the units, cut-off points, or categories of the results of the a) index test and  b) reference standard. | **Yes □ No □ Unclear □ N/A □** |
|  | **10** | Describe the number, training and expertise of the persons executing and reading the a) index tests and b) reference standards. | **Yes □ No □ Unclear □ N/A □** |
|  | **11** | Were the readers of the a) index test and b) reference standards blind (masked) to the results of the other test? Describe any other clinical information available to the readers. | **Yes □ No □ Unclear □ N/A □** |
| **Statistical Methods** | **12** | Describe methods for calculating or comparing methods of diagnostic accuracy and the statistical methods used to quantify uncertainty (e.g. 95% CI) | **Yes □ No □ Unclear □ N/A □** |
|  | **13** | Describe methods for calculating test reproducibility, if done. | **Yes □ No □ Unclear □ N/A □** |
| **RESULTS** | | | |
| **Participants** | **14** | Report when study was done, including beginning and ending dates of recruitment | **Yes □ No □ Unclear □ N/A □** |
|  | **15** | Report clinical and demographic characteristics of the study population (e.g. age, sex, spectrum of presenting symptoms, co morbidity, current treatments, recruitment centres) | **Yes □ No □ Unclear □ N/A □** |
|  | **16** | Report the number of participants satisfying the criteria for inclusion that did or did not undergo the index tests and/or the reference standard; describe why participants failed to receive either test. | **Yes □ No □ Unclear □ N/A □** |
| **Test results** | **17** | Report time interval from the index tests to the reference standard, and any treatment administered between. | **Yes □ No □ Unclear □ N/A □** |
|  | **18** | Report distribution of severity of disease (define criteria) in those with the target condition; other diagnoses in participants without the target condition. | **Yes □ No □ Unclear □ N/A □** |
|  | **19** | Report a cross tabulation of the results of the index tests (including indeterminate and missing results) by the results of the reference standard; for continuous results, the distribution of the test results by the results of the reference standard. | **Yes □ No □ Unclear □ N/A □** |
|  | **20** | Report any adverse events form performing the index tests or the reference standard. | **Yes □ No □ Unclear □ N/A □** |
| **Estimates** | **21** | Report estimates of diagnostic accuracy and measures of statistical uncertainty (e.g. 95% CI) | **Yes □ No □ Unclear □ N/A □** |
|  | **22** | Report how indeterminate results, missing responses and outliers of the index tests were handled. | **Yes □ No □ Unclear □ N/A □** |
|  | **23** | Report estimates of variability of diagnostic accuracy between subgroups of participants, readers or centres, if done. | **Yes □ No □ Unclear □ N/A □** |
|  | **24** | Report estimates of test reproducibility, if done. | **Yes □ No □ Unclear □ N/A □** |
| **DISCUSSION** |  |  |  |
|  | **25** | Discuss the clinical applicability of the study findings. | **Yes □ No □ Unclear □ N/A □** |
